# Resistance is futile: lack of predator switching and a preference for native prey predict the success of an invasive prey species

**DOI:** 10.1098/rsos.180339

**Published:** 2018-08-01

**Authors:** Ross N. Cuthbert, James W. E. Dickey, Clare McMorrow, Ciaran Laverty, Jaimie T. A. Dick

**Affiliations:** Institute for Global Food Security, School of Biological Sciences, Queen's University Belfast, Medical Biology Centre, 97 Lisburn Road, Belfast BT9 7BL, UK

**Keywords:** invasive species, success, prey switching, functional response, predation, biotic resistance

## Abstract

Invasive species continue to severely impact biodiversity, yet predicting the success or failure of introduced species has remained elusive. In particular, the relationship between community invasibility and native species diversity remains obscure. Here, we apply two traditional ecological concepts that inform prey population stability and hence invasibility. We first show that the native predatory crustacean *Gammarus duebeni celticus* exhibited similar type II (destabilizing) functional responses (FRs) towards native mayfly prey and invasive amphipod prey, when these prey species were presented separately. However, when the two prey species were presented simultaneously, the predator did not exhibit prey switching, instead consuming disproportionately more native prey than expected from the relative abundance of native and invasive species. These consumptive propensities foster reductions of native prey, while simultaneously limiting biotic resistance against the invasive species by the native predator. Since our theoretical considerations and laboratory results match known field invasion patterns, we advocate the increased consideration of FR and prey switching studies to understand and predict the success of invasive species.

## 1. Introduction

Invasive alien species present a continuing global threat to biodiversity, with the rate of invasions continuing to increase [1]. Debate continues surrounding the relationship between community invasibility and levels of ‘biotic resistance’ provided by community diversity [2], with a distinct lack of methodologies that can predict the success or failure of invasions. Here, we consider invasion success as the ability of an invader to establish, spread and reproduce in a novel environment (see [3]). Indeed, invasion science has been slow to develop truly predictive methods for invasion success and has often neglected to incorporate traditional ecological concepts [4]. Unifying such concepts across taxa and trophic groups and developing methods to quantify and better understand invasive species success are thus central to protection of biodiversity and ecosystem structure and function [5]. In particular, predicting the strength of biotic resistance by recipient communities to new invaders might inform strategies to mitigate invasion impacts.

Traditionally, ecologists have used the ‘functional response’ (FR; relationship between resource supply and resource use) to quantify interaction strengths, such as between predator and prey [6]. While this has seen some success in explaining invasions, there has been no incorporation of prey switching, also known as frequency-dependent predation [7], into predictive methods for invasion success. This is critical, as patterns of prey switching and prey preferences by predators have implications for the stability of prey populations and hence the degree of biotic resistance that community members exert on invasive species. That is, by regulating abundant prey while providing refuge for rare prey at low prey densities, prey switching may contribute to type III population stabilizing FRs, thus facilitating prey persistence [8]. Alternatively, where there are strong prey preferences and lack of prey switching behaviour, predators may severely lower the abundance of one prey species while facilitating expansion of another. In the context of invasion ecology, then, the latter scenarios could help predict if an invasive species would encounter high biotic resistance, and hence fail, or low biotic resistance, and hence invade successfully.

In this study, a common field pattern is examined, where an invasive amphipod, *Crangonyx pseudogracilis*, invades species-rich freshwater habitats, but faces predation by resident native amphipods [9,10]. A pattern of invasion success is theoretically likely, and hence predictable, if the invader suffers low biotic resistance from the native predator due to lack of prey switching and high preference for native prey, thus facilitating the invader. Here, we thus compare the FRs, prey switching and prey preferences of the native predatory amphipod *Gammarus duebeni celticus* to larvae of the native mayfly *Baetis rhodani* and the invasive gammarid *C. pseudogracilis*.

## 2. Material and methods

In November 2014, unparasitized male native river shrimp *G. d. celticus* (1.5–2 cm body length) were collected from Glen Road stream, County Down, UK (54.508° N, 5.9708° W). Their commonly consumed prey [11], native mayfly *B. rhodani* (0.7–0.8 cm) were collected from Dunore stream, County Antrim (54.680° N, 6.225° W), and the invasive amphipod *C. pseudogracilis* (0.7–0.9 cm) from ponds in Clandeboye Estate, County Down (54.641° N, 5.7139° W). Each species was transported to the Queen's University Belfast laboratory in source water and maintained separately in continuously aerated source water with stream flora and fauna supplied ad libitum at 12°C (±2°C) and a 12 L : 12 D regime.

*Gammarus duebeni celticus* were then selected haphazardly and starved individually for 24 h in cylindrical arenas of 8 cm diameter with 50 ml of filtered source water. In Experiment 1, for the two prey species separately, five prey densities (2, 4, 8, 16 and 32; *n* = 3 replicates per density) were introduced into arenas as above containing 150 ml of water and allowed to settle for 30 min prior to the addition of individual predators which were allowed to feed for 24 h; live and eaten prey were then counted. In Experiment 2, the two prey species combined were presented to individual predators for 3 h at seven prey species ratios (2 : 28, 4 : 26, 8 : 22, 15 : 15, 22 : 8, 26 : 4, 28 : 2; *n* = 6 replicates per ratio). Prey were replaced as they were consumed to maintain nominal prey species ratios. Controls for both experiments were one replicate of each experimental group with the predator absent (that is, 167 *B. rhodani* and 167 *C. pseudogracilis*).

Data analyses were undertaken in ‘R’. The package ‘frair’ was used for FR analyses [12]. In Experiment 1, logistic regression was used to infer FR forms, whereby a type II response is indicated by a significantly negative first-order term. To account for prey depletion, we fitted Rogers' random predator equation [13]:

2.1 $$N_{e}=N_{0}(1-\exp(a(N_{e}h-T))),$$

where *N*_e_ is the number of prey eaten, *N*_0_ is the initial density of prey, *a* is the attack constant, *h* is the handling time and *T* is the total experimental period. The Lambert W function was used for model fitting [14]. Data were non-parametrically bootstrapped (*n* = 2000) to generate 95% confidence intervals. In Experiment 2, Chesson's selectivity index [15,16] assuming prey replacement was used to determine preferences between prey across the varying prey proportions:

2.2 $$\alpha_{i}=\frac{r_{i}/n_{i}}{\overset{m}{\underset{j=1}{\sum }}(r_{j}/n_{j})},$$

where *α_i_* is Chesson's selectivity index for prey type *i*, *n_i_* is the number of prey type *i* available at the start of the experiment, *r_i_* is the number of prey type *i* consumed, *m* the number of prey types, *r_j_* is the number of prey type *j* consumed and *n_j_* the number of prey type *j* available at the start of the experiment. The value of *α_i_* ranges from 0 to 1, with 0 indicating complete avoidance and 1 indicating complete preference. In the two prey systems, values of 0.5 are indicative of no selectivity. Chesson's indices were transformed to reduce extremes (0s, 1s):

2.3 $$\alpha_{t}=\frac{\alpha_{i}(n-1)+0.5}{n},$$

where *α*_*t*_ is the transformed output and *n* is the sample size. Beta-regression using the ‘betareg’ package [17] in ‘R’ was used to compare *α*_*t*_ values between ‘prey’ and ‘proportion’, and their interaction.

## 3. Results

**Figure 1.**
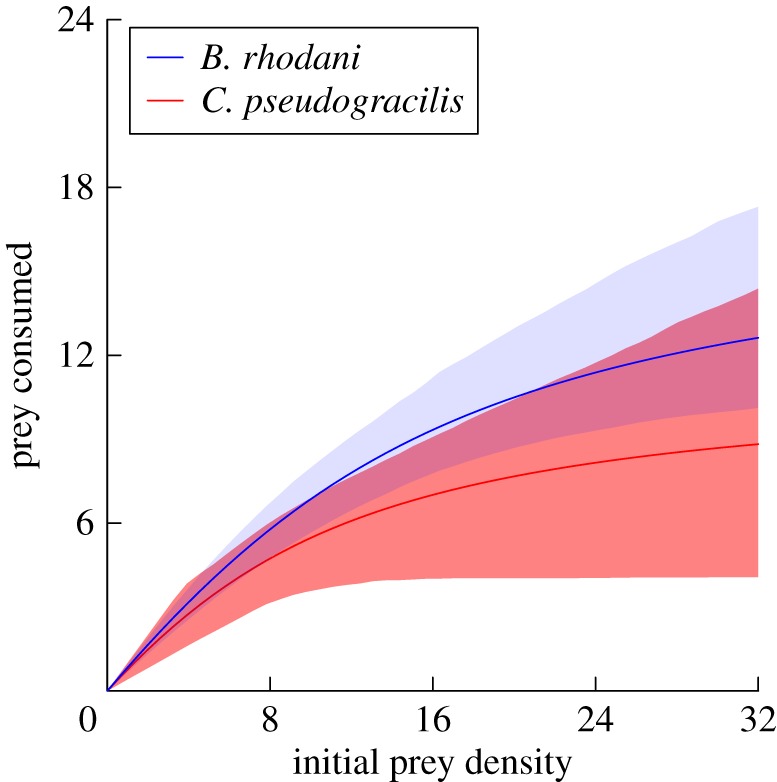
FRs of the native predator *G. d. celticus* towards native *B. rhodani* and invasive *C. pseudogracilis* prey with bootstrapped (*n* = 2000) 95% CIs.

**Figure 2.**
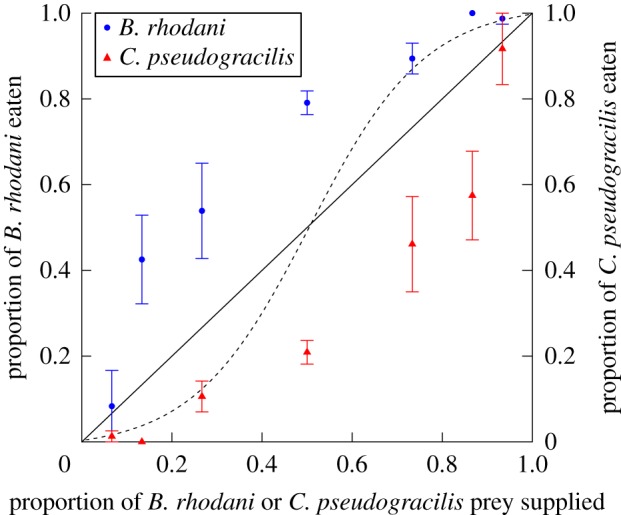
Proportion of either *B. rhodani* or *C. pseudogracilis* in the diet of *G. d. celticus* as a function of the proportion of each prey species supplied. The solid line indicates the expected values if there was no preference between the two prey types. The dashed sigmoid line represents a hypothetical prey switching pattern. Means are ±s.e. (*n* = 6).

Survival in controls was 100% in both experiments. Thus, prey mortality was attributed entirely to predation, which was also observed directly. In Experiment 1, destabilizing type II FRs were detected for predation on each prey species (*B. rhodani*, first-order term = −0.07, *p* < 0.001; *C. pseudogracilis*, first-order term = −0.05, *p* < 0.001). Functional response magnitude (curve asymptote) trended towards being higher upon the native *B. rhodani*; however, confidence intervals overlapped across all prey densities, and thus, similarities for prey in attack rate and handling time parameters can be deduced (figure 1). In Experiment 2, however, significantly disproportionately more *B. rhodani* were consumed by *G. d. celticus* at all prey proportions, indicating a lack of switching between prey and consistent preferential predation of the native *B. rhodani* over the invasive *C. pseudogracilis* (figure 2). Chesson's indices indicated a significant preference for *B. rhodani* over *C. pseudogracilis* overall (*χ*^2^ = 59.29, d.f. = 1, *p* < 0.001). Prey preferences were significantly affected by proportions available (*χ*^2^ = 64.50, d.f. = 6, *p* < 0.001), and there was a significant ‘prey × proportion’ interaction (*χ*^2^ = 20.20, d.f. = 6, *p* = 0.003) reflecting increases in preference towards *B. rhodani* at intermediate prey proportions (figure 2).

## 4. Discussion

The impacts of invasive species on native species populations can be severe and are continuing, and thus, the development of predictive methodologies is crucial to effectively understand and forecast invader success or failure in terms of establishment and spread. Furthermore, we require elucidation of why there are no consistent patterns of invasibility with respect to community diversity. The use of FRs has provided meaningful insights in the context of invader impact [5], particularly for predators, but examining just single-prey species obscures effects on prey populations mediated through prey switching, or lack thereof [7]. Furthermore, while there are other biotic interactions, such as parasitism [18] and competition [19], that can have profound effects on community interaction outcomes, FRs are also able to quantify such outcomes [4,5]. Here, *G. d. celticus* exhibited potentially population destabilizing type II FRs towards two prey species, with similarities in consumption (attack rates and handling times) of both prey species when presented separately. However, where the two prey species were supplied to the predator simultaneously, no prey switching occurred across the prey ratio spectrum, with *G. d. celticus* consistently showing a significant preference for the native *B. rhodani* over the invasive *C. pseudogracilis*. The biotic resistance hypothesis posits that non-native species can be prevented from establishing and spreading, and hence exerting negative ecological impact, due to resident predators and competitors in the recipient area, with higher species richness conducive to higher resistance [20]. However, we demonstrate that *C. pseudogracilis* populations may be alleviated of resistance by predators which disproportionately consume higher levels of native prey across relative prey availabilities. This, coupled with the type II FR demonstrated, may drive localized reductions/extinctions of native species and hence facilitation of invasive species. Indeed, mayfly populations are known to be seriously depleted in the face of predation by *Gammarus* species [11,21] and *C. pseudogracilis* invades species-rich aquatic systems [9,10]. Our current study thus corroborates theory with field patterns and hence shows predictability of invasion success where native prey items are also present.

In summary, the application of FRs, prey switching and prey preferences to invasion ecology can foster a new framework to better understand biotic resistance and invader success. While the comparative FR method has been shown repeatedly to be a robust predictor of invader impact [5], shortcomings exist with regard to its use in testing biotic resistance in single-prey studies. We propose that the study of prey switching and preference alongside FRs under different environmental contexts is crucial to derive a more holistic account of the success of invaders and hence impacts on native prey populations, alongside the capacity for biotic resistance by recipient communities.

## Supplementary Material

Functional response data

## Supplementary Material

Prey switching data

## Data accessibility

Raw data can be found in the electronic supplementary material**.**
